# Atezolizumab Plus Bevacizumab for Advanced Hepatocellular Carcinoma with Macroscopic Vascular Invasion: An Inverse Probability of Treatment Weighted Analysis

**DOI:** 10.3390/cancers18010033

**Published:** 2025-12-22

**Authors:** Jihoon Kim, Jin-Hyoung Kim, Byung Soo Im, Gun Ha Kim, Hee Ho Chu, Dong Il Gwon, Ji Hoon Shin, Ju Hyun Shim, Sang Min Yoon, Sehee Kim

**Affiliations:** 1 Department of Radiology, Asan Medical Center, University of Ulsan, Seoul 05505, Republic of Korea; jihoonkim@amc.seoul.kr (J.K.); kofi@amc.seoul.kr (B.S.I.); kimgh.rad@amc.seoul.kr (G.H.K.); d180717@amc.seoul.kr (H.H.C.); radgwon@amc.seoul.kr (D.I.G.); jhshin@amc.seoul.kr (J.H.S.); 2 Department of Gastroenterology, Asan Medical Center, University of Ulsan College of Medicine, Seoul 05505, Republic of Korea; s5854@amc.seoul.kr; 3 Department of Radiation Oncology, Asan Medical Center, University of Ulsan College of Medicine, Seoul 05505, Republic of Korea; drsmyoon@amc.seoul.kr; 4 Department of Clinical Epidemiology and Biostatistics, University of Ulsan College of Medicine, Seoul 05505, Republic of Korea; sehee_kim@amc.seoul.kr

**Keywords:** hepatocellular carcinoma, macrovascular invasion, atezolizumab plus bevacizumab, locoregional therapy

## Abstract

**Background/Objectives**: Management of hepatocellular carcinoma (HCC) with macrovascular invasion (MVI) varies between systemic immunotherapy and locoregional approaches. We compared atezolizumab plus bevacizumab (Atezo–Bev) with locoregional therapy in treatment-naïve patients. **Methods**: We conducted a retrospective cohort study of patients with image- or biopsy-proven HCC and MVI, Child–Pugh A/B, and ECOG 0–1 who received first-line Atezo–Bev or locoregional therapy (transarterial chemoembolization [TACE] with or without external-beam radiotherapy [RT]). Inverse probability of treatment weighting (IPTW) minimized baseline imbalances. Primary outcomes were overall survival (OS) and progression-free survival (PFS). Modified RECIST assessed radiologic response, and major adverse events were classified using Society of Interventional Radiology criteria. **Results**: We analyzed 475 patients (Atezo–Bev, *n* = 191; locoregional therapy, *n* = 284). Baseline characteristics were similar, and IPTW achieved covariate balance. Median OS was 9.3 months with Atezo–Bev and 10.8 months with locoregional therapy; after IPTW, OS remained comparable (hazard ratio [HR] 0.95; 95% CI 0.76–1.19; *p* = 0.635). Median PFS was 6.0 versus 4.1 months, favoring Atezo–Bev; this persisted after IPTW (HR 0.64; 95% CI 0.52–0.79; *p* < 0.001). Objective response rates were similar (45% vs. 48%; *p* = 0.49). Major adverse events occurred in 11% of patients in both groups. Subgroup analyses showed no OS differences and a consistent PFS advantage with Atezo–Bev. **Conclusions**: In HCC with MVI, first-line Atezo–Bev achieved longer PFS than locoregional therapy, with comparable OS and safety, supporting Atezo–Bev as a valid and effective first-line option for disease control while locoregional modalities remain relevant within multidisciplinary care.

## Simple Summary

Patients with liver cancer that grows into major blood vessels typically have a poor outlook and limited treatment options. Clinicians often choose between two very different approaches: a modern drug combination (atezolizumab plus bevacizumab) or locoregional treatments such as targeted chemotherapy delivered into the liver, sometimes combined with radiation. However, it has not been clear which option works better for these high-risk patients. In this study, we examined real-world outcomes from nearly 500 patients to compare these two strategies fairly. We found that the drug combination delayed cancer progression for a longer period, while overall survival and rates of serious side effects were similar between the two approaches. These findings provide practical evidence to help guide treatment decisions and support more personalized care for patients with advanced liver cancer involving major blood vessels.

## 1. Introduction

Treatment of advanced hepatocellular carcinoma (HCC) remains a major clinical challenge. Despite advances in surveillance and imaging, more than half of patients are still diagnosed at intermediate or advanced stages, when curative options are rarely feasible and prognosis is poor. The presence of macrovascular invasion (MVI) further limits therapeutic choices and worsens outcomes [1,2,3]. Current international guidelines recommend systemic therapy as the standard of care for HCC with MVI, with the 2022 update of the Barcelona Clinic Liver Cancer (BCLC) strategy identifying immunotherapy-based regimens as first-line options [4,5].

Among available regimens, atezolizumab plus bevacizumab (Atezo–Bev) has become the preferred first-line therapy based on the IMbrave150 trial, which demonstrated significant improvements in overall survival (OS) and progression-free survival (PFS) compared with sorafenib, supported by updated subgroup analyses. This demonstrable clinical superiority led the BCLC 2022 staging system update to designate immunotherapy-based regimens as the new first-line standard for advanced HCC, fundamentally shifting the treatment paradigm away from solely tyrosine kinase inhibitors [4,5]. The robust efficacy of this regimen is attributed to a synergistic mechanism: bevacizumab’s inhibition of VEGF not only limits tumor angiogenesis but also re-models the immunosuppressive tumor microenvironment, thereby enhancing T-cell infiltration and the anti-tumor activity of atezolizumab’s PD-L1 blockade [6,7,8]. Subsequent studies have confirmed its efficacy and manageable safety profile across diverse populations [9,10,11].

In real-world practice, locoregional therapy—defined as transarterial chemoembolization (TACE) with or without external-beam radiotherapy (RT)—continues to play a major role for patients with MVI, particularly in East Asia [12,13]. A randomized clinical trial showed that TACE combined with RT (TACE + RT) achieved superior OS and PFS compared with sorafenib, underscoring its potential benefit in selected patients [14]. These results have influenced regional guidelines, which now recommend both systemic immunotherapy and selected locoregional combinations for patients with vascular invasion [15].

However, direct comparative data between Atezo–Bev and contemporary locoregional therapy for HCC with MVI remain limited. A recent multicenter study compared outcomes of Atezo–Bev with TACE + RT in patients with portal vein tumor thrombosis (PVTT); however, its scope was restricted to that subgroup and did not address the broader MVI spectrum. Therefore, this study aimed to evaluate whether Atezo–Bev provides superior survival and disease control compared with locoregional therapy in treatment-naïve patients with unresectable HCC and MVI, using propensity score weighting to minimize baseline imbalance [16].

## 2. Materials and Methods

### 2.1. Study Eligibility

This study was approved by the Institutional Review Board of Asan Medical Center (approval No. 2024-1408) on 25 November 2024, which waived the requirement for patient consent due to its retrospective design. Inclusion criteria were image- or biopsy-confirmed HCC based on recent international guidelines [17,18], HCC with MVI, Child–Pugh class A or B liver function, and an Eastern Cooperative Oncology Group (ECOG) performance status of 0–1. Exclusion criteria were applied to ensure patient safety and to minimize confounding variables in this comparative retrospective analysis. These included Child–Pugh class C liver function (due to the heightened risk of drug-related toxicity and poor prognosis), total serum bilirubin ≥ 3 mg/dL (indicating severe hepatic dysfunction), tumor involvement of more than half of the liver (due to extremely poor prognosis and high risk of hepatic failure), or ECOG performance status 3–4 (as these patients are generally unable to tolerate aggressive first-line therapies and poor performance status is a strong confounding factor for survival outcomes). Patients who received Atezo–Bev or locoregional therapy (cisplatin-based TACE delivered through the hepatic artery, with or without external-beam RT directed to the MVI) as first-line treatment for treatment-naïve HCC with MVI were included. Between November 2020 and June 2024, 191 treatment-naïve patients with HCC were among 284 treatment-naïve patients with HCC and MVI who received first-line TACE + RT or TACE alone between January 2018 and October 2020.

### 2.2. Atezo–Bev Treatment

Patients received bevacizumab 15 mg/kg and atezolizumab 1200 mg intravenously every 3 weeks, following the IMbrave150 trial protocol [6,8]. Dose adjustments or permanent discontinuation of either agent were performed primarily based on the occurrence of adverse events graded according to the Society of Interventional Radiology (SIR) Classification System for Complications. Specifically, treatment was interrupted or permanently discontinued for Grade 3 or higher adverse events that were considered treatment-related and clinically unmanageable, following institutional guidelines [19,20]. Therapy continued until patients experienced unacceptable toxicity or disease progression [12].

### 2.3. TACE

Cisplatin-based TACE was performed using a cisplatin dose of 2 mg/kg. Through a microcatheter, 4–20 mL of a 1:1 emulsion of cisplatin and iodized oil (Lipiodol^®^, Guerbet, Roissy, France) was infused into the lobar, segmental, or subsegmental feeding artery, followed by embolization with Gelfoam slurry (Upjohn, Kalamazoo, MI, USA) until arterial flow was nearly stagnant [20]. In patients with significant arteriovenous or arterioportal shunts, initial embolization with Gelfoam slurry was performed to occlude the shunt, followed by infusion of the iodized oil/cisplatin emulsion and additional Gelfoam embolization [21]. For patients with main PVTT and poorly developed collaterals, transarterial chemotherapeutic infusion without Gelfoam was performed [22].

TACE was repeated every 4–6 weeks if residual viable tumor tissue was detected on follow-up CT without deterioration of hepatic function. In some patients (*n* = 29), only TACE was performed because of limited tumor thrombus extent or refusal of additional RT. TACE alone was also performed when EBRT was not feasible because of liver reserve, anatomic limitations, prior radiation, or patient refusal. These indications reflect institutional practice standards during the study period.

### 2.4. External-Beam RT

RT methods at our institution have been described previously [22,23]. Patients began RT for vascular invasion within 3 weeks of the initial TACE. Four-dimensional CT simulation (GE Healthcare, Waukesha, WI, USA) was performed during free breathing in all patients. The synchronized four-dimensional CT images were divided into 10 series according to respiratory phase (Advantage 4D version 4.2; GE Healthcare, Waukesha, WI, USA). Respiratory motion was tracked using a real-time position management gating system (Varian Medical Systems, Palo Alto, CA, USA).

Gross tumor volume (GTV) for large, multiple, or infiltrative HCCs included the tumor thrombus and a 2 cm margin into contiguous HCC at end-expiration. For small HCCs encompassed within a single radiation port, GTV included the entire tumor and thrombus [22,23]. The internal target volume (ITV) was defined as the sum of all GTVs across gated respiratory phases, and the planning target volume (PTV) was defined as the ITV plus 0.7 cm margins [22,23].

Target volumes, radiation ports, and doses were determined using a three-dimensional conformal planning system (Eclipse version 10.0, Varian Medical Systems, Palo Alto, CA, USA). Radiation was delivered via a respiratory-gated beam technique, with a total dose of 45 Gy in 2–5 Gy fractions, five times per week, using 6-, 10-, or 15-MV X-rays on a linear accelerator (Varian Medical Systems, Palo Alto, CA, USA) [22,23].

### 2.5. Radiologic Response Assessment

Radiologic responses were assessed every 4–6 weeks using dynamic CT or MRI according to the modified Response Evaluation Criteria in Solid Tumors (mRECIST) guidelines [24]. Responses were classified as objective response (complete response [CR] or partial response [PR]) or non-regression (stable disease [SD] or progressive disease [PD]) [25]. The best overall response observed during treatment was recorded as the final response [26].

### 2.6. Evaluation of Adverse Events

Major adverse events following Atezo–Bev or locoregional therapy were evaluated according to the SIR reporting standards [20]. Adverse events were considered treatment-related if they occurred during or within 90 days after the end of treatment and were deemed by the treating physician to be potentially attributable to the administered agent or procedure. The SIR defines major adverse events as those requiring additional treatment, prolonged hospitalization, increased level of care, substantial morbidity, or resulting in death (SIR classifications grade 3–5).

### 2.7. Definitions and Data Analysis

Comparisons between the two treatment groups were performed using the independent t-test for continuous variables and the *χ*^2^ test for categorical variables. PFS and OS were calculated using the Kaplan–Meier method and compared using log-rank tests. PFS was defined as the interval from treatment initiation to tumor progression or death from any cause. OS was defined as the interval from initial treatment to death from any cause.

Survival data were censored when patients underwent surgical resection or liver transplantation after receiving Atezo–Bev or locoregional therapy, who achieved successful tumor downstaging following the initial treatment, making them eligible for curative therapy despite initial MVI, as these interventions could substantially alter outcomes. Timepoint-specific Kaplan–Meier estimates are provided in Table S1.

### 2.8. Propensity Score Weighting

To minimize confounding by indication between treatment groups, inverse probability of treatment weighting (IPTW) was applied. Propensity scores—representing the probability of receiving Atezo–Bev—were estimated using multivariable logistic regression incorporating clinically relevant covariates: age, etiology of liver disease, Child–Pugh class, MVI, extrahepatic metastasis, maximum tumor size, tumor multiplicity, and serum alpha-fetoprotein (≥400 ng/mL).

Stabilized weights were computed and truncated to reduce the influence of extreme 10.8 differences, with <0.10 indicating adequate balance. Time-to-event outcomes were analyzed using IPTW-weighted Cox proportional hazards models, and hazard ratios (HRs) with 95% confidence intervals (CIs) were reported.

All tests were two-sided, with statistical significance set at *p* < 0.05. Analyses were performed using SAS version 9.4 (SAS Institute, Inc., Cary, NC, USA) and R version 4.4.2 (R Foundation for Statistical Computing, Vienna, Austria).

## 3. Results

### 3.1. Patient Characteristics

Table 1 presents the baseline demographic and clinical characteristics of both groups. All patients had radiologically confirmed MVI on contrast-enhanced CT or MRI and were therefore classified as BCLC stage C according to the 2022 BCLC guidelines. The median size of the largest tumor was 10 cm (interquartile range [IQR], 7–14 cm). Most patients in both groups were male and had chronic hepatitis B virus infection. No significant differences were observed between the groups regarding age, sex, etiology, Child–Pugh class, tumor burden (maximum tumor size and tumor number), elevated serum alpha-fetoprotein levels, tumor type (nodular or infiltrative), or extent of vascular invasion. The presence of a numerical imbalance in the most prognostically adverse factor, Main PV or IVC invasion (Atezo–Bev: 56.0% vs. LRT: 62.3%; *p* = 0.17), a clinically critical variable, was specifically addressed by the IPTW adjustment, which achieved adequate covariate balance for this factor (ASD < 0.1). In addition, significant differences were noted in ECOG performance status (*p* = 0.001), presence of varices (*p* < 0.001), and extrahepatic metastasis (*p* = 0.003). After IPTW adjustment, covariate balance was achieved, with all absolute standardized mean differences < 0.1.

### 3.2. OS and PFS Analyses

By the end of follow-up, 146 patients (76%) in the Atezo–Bev group and 261 (92%) in the locoregional therapy group had died. No patients in the Atezo–Bev group and 18 patients in the locoregional therapy group were censored at the time of surgical resection or liver transplantation.

**Figure 1:**
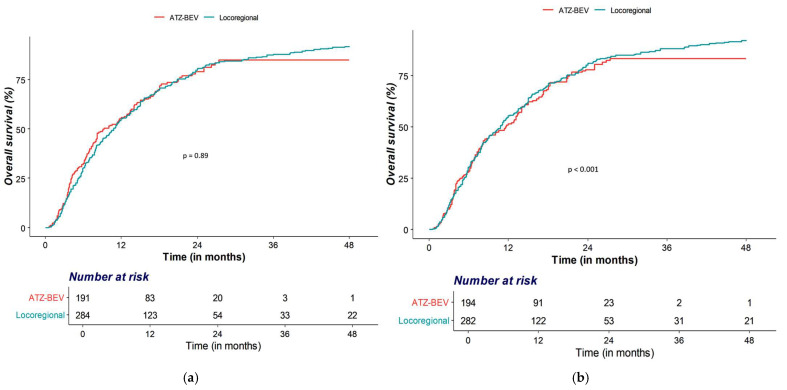
Kaplan–Meier curves before propensity score analysis by treatment group. (**a**) unadjusted overall survival, (**b**) unadjusted progression-free survival.

The OS rates at 6 months and at 1, 2, 3, and 4 years were 68%, 44%, 21%, 15%, and 15%, respectively, in the Atezo–Bev group, and 70%, 45%, 19%, 12%, and 8%, respectively, in the locoregional therapy group. Median OS did not differ significantly between the Atezo–Bev group (9.3 months) and the locoregional therapy group (10.8 months) (*p* = 0.89) (Figure 1a).

During follow-up, 160 patients (84%) in the Atezo–Bev group and 265 (93%) in the locoregional therapy group experienced disease progression or death. The PFS rates at 6 months and at 1, 2, 3, and 4 years were 50%, 29%, 15%, 10%, and 10%, respectively, for Atezo–Bev, and 40%, 16%, 6%, 5%, and 3%, respectively, for locoregional therapy. Median PFS was significantly longer with Atezo–Bev (6.0 months) than with locoregional therapy (4.1 months; *p* < 0.001) (Figure 1b).

**Figure 2:**
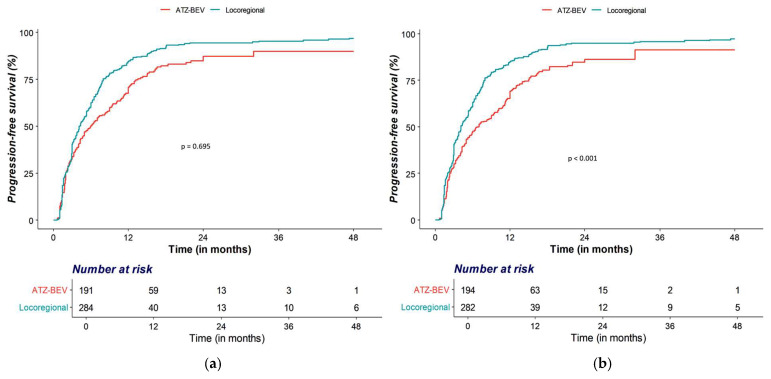
Kaplan–Meier curves after propensity score analysis by treatment group. (**a**) IPTW-weighted overall survival, (**b**) IPTW-weighted progression-free survival.

After IPTW, the OS difference remained non-significant (HR 0.95; 95% CI 0.76–1.19; *p* = 0.635) (Figure 2a), whereas PFS consistently favored Atezo–Bev (HR 0.64; 95% CI 0.52–0.79; *p* < 0.001) (Figure 2b) (Table 2).

### 3.3. Subgroup Analyses

**Figure 3:**
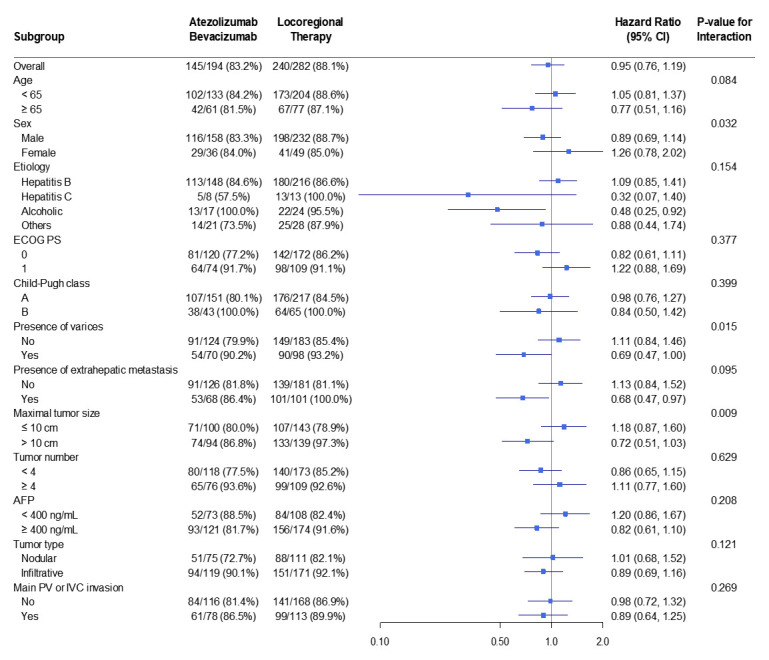
Forest plot of the 3-year overall survival in the IPTW-weighted sample.

In prespecified subgroup analyses, the treatment effect on 3-year OS (Figure 3) was consistent with the primary analysis. Stratification by age, etiology of liver disease, Child–Pugh class, MVI, extrahepatic metastasis, maximum tumor size, and tumor multiplicity produced HRs clustering around unity, with no subgroup-specific differences. These findings were similar in both unadjusted and IPTW-weighted models, reinforcing the robustness of the null OS result.

**Figure 4:**
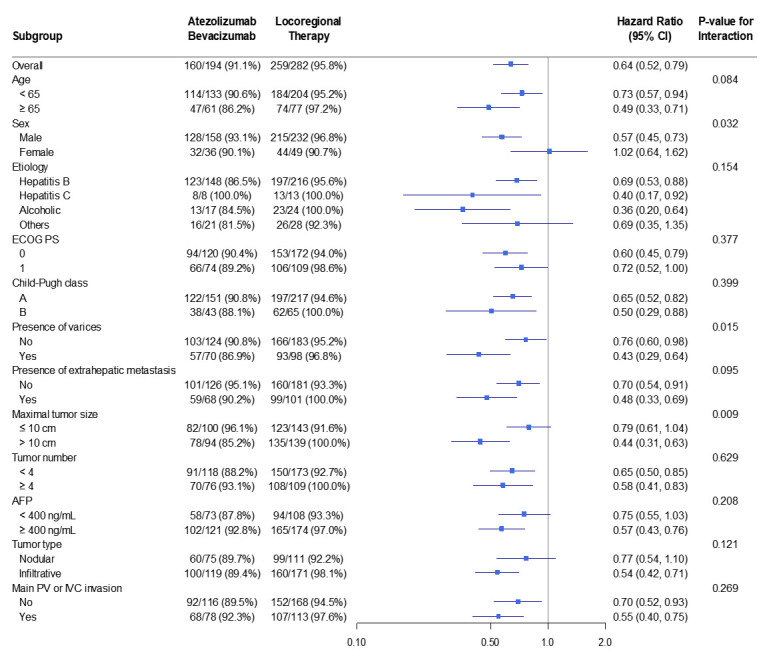
Forest plot of the 3-year progression-free survival in the IPTW-weighted sample.

Conversely, in the 3-year PFS analysis (Figure 4), Atezo–Bev demonstrated a consistent advantage over locoregional therapy across subgroups. Point estimates favored Atezo–Bev-weighted result. Unadjusted analyses showed similar trends, indicating that the PFS benefit was broadly applicable rather than confined to a specific subgroup.

### 3.4. Radiologic Response After Treatment

During follow-up, 16 patients (8%) in the Atezo–Bev group achieved CR, 70 (37%) achieved PR, 55 (29%) showed PD, and 50 (26%) had SD. In the locoregional therapy group, 43 (15%) and 94 (33%) achieved CR and PR, respectively, while 81 (28%) showed PD and 66 (23%) had SD. The objective tumor response rate (CR or PR) did not differ significantly between the Atezo–Bev and locoregional therapy groups (45% vs. 48%) (*p* = 0.49) (Table 3). After IPTW adjustment, the incidence of objective tumor response remained similar (48% vs. 48%, *p* = 0.96).

### 3.5. Major Adverse Events

Major adverse events occurred in 21 (11%) of the 191 patients receiving Atezo–Bev and in 32 (11%) of the 284 patients receiving locoregional therapy (*p* = 0.93) (Table 3). In the Atezo–Bev group, major adverse events included proteinuria (*n* = 4), bleeding (*n* = 4), drug-related hepatitis (*n* = 3), skin rash (*n* = 3), drug-related pneumonitis (*n* = 2), severe diarrhea (*n* = 2), drug-related pancreatitis (*n* = 1), drug-induced adrenal insufficiency (*n* = 1), and toxic epidermal necrolysis (*n* = 1). In the locoregional therapy group, adverse events included acute kidney injury (*n* = 9), hepatic failure (*n* = 6), ischemic cholangiopathy with biloma (*n* = 5), liver abscess (*n* = 3), cisplatin-related allergic reaction (*n* = 3), cisplatin-induced neuropathy (*n* = 2), tumor lysis syndrome (*n* = 1), spinal cord infarction (*n* = 1), pneumonia (*n* = 1), and RT-related colitis (*n* = 1). After IPTW adjustment, the incidence of major adverse events remained comparable between the groups (11.4% vs. 11.4%; *p* = 0.99).

## 4. Discussion

To our knowledge, this is the first propensity score–weighted comparative analysis of Atezo–Bev versus locoregional therapy in patients with HCC and MVI. PFS was significantly longer with Atezo–Bev, whereas OS was comparable between groups. These findings were reproduced in inverse probability weighting analyses, underscoring robustness against selection bias. Major adverse events occurred at similar frequencies for both treatment strategies according to SIR criteria, before and after IPTW adjustment.

Although current guidelines recommend Atezo–Bev as the first-line regimen for advanced HCC [4,18], real-world management remains heterogeneous, particularly among patients with MVI, in whom locoregional therapy continues to be used in selected candidates [27,28]. In this study, Atezo–Bev yielded longer PFS than locoregional therapy, while OS did not differ significantly. The observed PFS benefit likely reflects improved disease control during active therapy, whereas OS convergence may result from subsequent treatments and post-progression crossover, which often obscure survival differences between regimens. Moreover, competing risks and extended post-progression survival can reduce the sensitivity of OS to on-treatment effects within a limited follow-up period [6,13,29,30]. In addition, post-progression and crossover treatments were heterogeneous and not recorded in sufficient detail to permit formal adjustment. This incomplete characterization of subsequent therapies likely contributed to OS convergence between groups and may partly explain the divergence between PFS and OS observed in the present study. Although the absolute median PFS gain of 1.9 months may appear modest, in patients with advanced HCC with MVI, this corresponds to a substantial relative reduction in the risk of progression or death and was generally accompanied by delayed clinical deterioration, including worsening liver function and performance status. Thus, we interpret the PFS benefit of Atezo–Bev as reflecting improved on-treatment disease control rather than a purely radiographic delay. Nevertheless, the lack of a demonstrable OS advantage indicates that the ultimate clinical impact of this PFS prolongation is attenuated by complex post-progression management and era-related factors.

Therefore, PFS may serve as a more responsive indicator of sustained disease control. The consistent PFS advantage with Atezo–Bev represents a clinically meaningful benefit, even in the absence of OS separation. This interpretation aligns with prior evidence showing weaker correlations between progression-based endpoints and OS in modern multi-line treatment settings, as well as randomized data confirming the disease-control capacity of Atezo–Bev [5,8]. Nonetheless, the lack of a detectable OS advantage indicates that the clinical impact of Atezo–Bev should be interpreted with caution. In this context, the PFS benefit is best viewed as enhanced on-treatment disease control, potentially translating into delayed symptomatic progression, prolonged maintenance of liver function, and more opportunities for subsequent therapies rather than as evidence of a clearly superior survival strategy. Thus, Atezo–Bev should be regarded as one of several viable first-line options, with locoregional therapy remaining an important alternative in appropriately selected patients. The comparable OS result also suggests that the locoregional therapy provided a robust survival benefit in this selected high-risk population. This observation aligns with existing regional data supporting the efficacy of TACE + RT combinations, which may effectively downstage or control local disease, thereby providing a durable OS foundation that is not easily surpassed by systemic therapy alone. Furthermore, the aggressive tumor biology inherently linked to macrovascular invasion represents a major confounding factor. This inherent aggressiveness may significantly limit the long-term survival window, causing OS convergence despite initial superior disease control achieved with Atezo–Bev.

Lee et al. reported superior PFS and OS with Atezo–Bev compared with TACE + RT in patients with PVTT without extrahepatic metastasis [16]. The modest discrepancies between their findings and the present results may reflect this study’s inclusion of a broader and more adverse disease spectrum, encompassing vascular invasion beyond the portal vein, such as hepatic venous and inferior vena caval involvement, and cases with multiple vascular territories affected [31,32]. Another contributing factor may be differences in intra-arterial chemotherapy protocols. In the current cohort, cisplatin was predominantly used, with limited application of doxorubicin. Cisplatin acts as a radiosensitizer by inhibiting DNA-dependent protein kinase–mediated nonhomologous end joining, thereby enhancing radiation-induced cytotoxicity, and has long served as a concurrent agent in definitive chemoradiation across tumor types [33,34]. These protocol-level variations may influence radiosensitization and intrahepatic cytoreduction, altering synergy with RT and potentially affecting OS. While the findings of Lee et al. provide important context, both their study and ours have inherent limitations, and therefore any cross-study comparison should be interpreted with caution.

The rate of major adverse events was 11.0% with Atezo–Bev and 11.3% with locoregional therapy, indicating comparable safety (*p* = 0.93). These rates align with published data reporting major adverse events in approximately 3–10% of patients receiving Atezo–Bev and 2–12% after locoregional therapy [6,7,8,14,35]. Given the similar safety profiles, locoregional therapy may remain a viable option in selected patients with HCC and MVI, particularly before systemic therapy initiation [18,36]. Moreover, strategically combining modalities may provide synergistic or additive disease-control benefits in appropriately selected candidates [14,34].

In the prespecified subgroup analyses, the treatment effect on OS was broadly consistent across clinical strata. HRs for age, etiology, liver function, tumor burden, and vascular invasion centered near unity, with substantial overlap of CIs, indicating similar survival between treatment strategies across patient groups. In contrast, for PFS, Atezo–Bev showed a reproducible advantage across nearly all subgroups. Most point estimates fell to the left of unity on the forest plot, suggesting a reduced risk of progression with Atezo–Bev. The direction and magnitude of benefit closely matched the overall treatment effect. Although CIs were wider in smaller subgroups, this reflected limited sample size rather than true heterogeneity. Collectively, the forest plots demonstrate that disease control with Atezo–Bev was consistently superior, while long-term survival remained comparable—reflecting both the biological activity of Atezo–Bev and the mitigating impact of effective locoregional therapy on mortality.

To improve outcomes beyond those achieved with Atezo–Bev in patients with HCC and MVI, ongoing research is exploring alternative strategies, particularly TARE. Observational studies comparing TARE with Atezo–Bev monotherapy have shown similar efficacy in some contexts. However, emerging analyses in PVTT suggest that, with optimized patient selection and dosimetry, TARE may outperform tyrosine kinase inhibitors and warrants direct comparison with Atezo–Bev in contemporary trials [37,38,39,40,41]. Kim et al. [38] proposed that earlier studies were limited by suboptimal selection—such as inclusion of main-trunk PVTT—and nonpersonalized, low-dose delivery. They advocated individualized high-dose TARE restricted to segmental or lobar portal vein involvement.

This study has some limitations. First, its retrospective design introduces potential selection bias and residual confounding despite statistical adjustment. To mitigate these risks, IPTW was applied, and balance diagnostics confirmed improved comparability of key baseline covariates between groups. Second, the comparison was partly historical, as Atezo–Bev was established as the first-line standard for advanced HCC only in 2020 [6,7,42]. Consequently, we acknowledge the inherent limitation that IPTW cannot fully adjust for unmeasured, time-varying confounders such as calendar-time effects, differences in follow-up, advances in supportive care or imaging, and critically, improvements in post-progression systemic treatments over the study period. These factors may have specifically influenced the survival outcome. Third, the locoregional therapy cohort was heterogeneous, including TACE alone and TACE combined with external-beam RT, with variations in intra-arterial chemotherapy and radiation delivery. This variability limits the ability to attribute observed effects to specific components of locoregional therapy and precludes detailed mechanistic interpretation of whether intra-arterial chemotherapy, embolization, radiotherapy, or their interaction primarily drives the observed outcomes. Accordingly, our findings should not be overinterpreted as comparative evidence for or against any specific TACE or TACE + RT protocol. Fourth, our cohort predominantly comprised patients with advanced disease characterized by extensive and heterogeneous MVI, including hepatic vein and inferior vena cava involvement, and frequent extrahepatic metastases. As a result, the external validity of our findings is limited, and they should not be directly extrapolated to more selected or earlier-stage patients with less extensive vascular involvement. Furthermore, we did not systematically capture detailed patterns of progression or time-to-response beyond standard mRECIST assessments in this retrospective cohort. As a result, we cannot fully delineate whether the PFS advantage of Atezo–Bev arose primarily from more durable intrahepatic control, delayed extrahepatic spread, or a reduction in early progression events. Future prospective multicenter randomized trials using standardized locoregional therapy protocols, predefined sequencing of systemic and locoregional modalities, and prespecified stratification by vascular invasion subtype and liver function, together with liver function preserving endpoints and optimized dosimetry, are needed to confirm these findings and to refine the optimal integration of Atezo–Bev and locoregional strategies.

## 5. Conclusions

In this IPTW-weighted analysis, Atezo–Bev demonstrated a clinically meaningful advantage in on-treatment disease control compared with locoregional therapy in patients with HCC and MVI. Nonetheless, locoregional therapy remains a relevant component of multidisciplinary care and may be incorporated into a staged treatment plan alongside Atezo–Bev for appropriately selected candidates. However, its precise role as a first-line option relative to Atezo–Bev remains to be defined and will ultimately need to be established in prospective randomized trials directly comparing systemic and locoregional strategies in well-stratified cohorts.

## Figures and Tables

**Table 1:** Baseline characteristics of the study population before and after IPTW.

Variables	Unadjusted				IPTW-Weighted			
	Atezolizumab–Bevacizumab (*n* = 191)	Locoregional Therapy (*n* = 284)	ASD	*p* *	Atezolizumab–Bevacizumab (*n* = 194)	Locoregional Therapy (*n* = 282)	ASD	*p* *
Age	58.98 (10.89)	57.96 (10.69)	0.095	0.311	58.55 (10.72)	58.20 (10.82)	0.033	0.745
Sex (%)			0.033	0.722			0.028	0.784
Male	157 (82.2)	237 (83.5)			158.0 (81.4)	232.4 (82.5)		
Female	34 (17.8)	47 (16.5)			36.1 (18.6)	49.3 (17.5)		
Etiology			0.231	0.108			0.028	0.994
HBV	139 (72.8)	223 (78.5)			148.0 (76.3)	216.3 (76.8)		
HCV	6 (3.1)	16 (5.6)			8.4 (4.3)	13.1 (4.7)		
Alcohol	20 (10.5)	20 (7.0)			16.6 (8.5)	23.7 (8.4)		
Others	26 (13.6)	25 (8.8)			21.0 (10.8)	28.5 (10.1)		
ECOG PS			0.337	<0.001			0.009	0.93
0	98 (51.3)	192 (67.6)			119.6 (61.6)	172.4 (61.2)		
1	93 (48.7)	92 (32.4)			74.4 (38.4)	109.2 (38.8)		
Child–Pugh class			0.055	0.56			0.022	0.827
A	149 (78.0)	215 (75.7)			150.9 (77.8)	216.5 (76.9)		
B	42 (22.0)	69 (24.3)			43.1 (22.2)	65.2 (23.1)		
Presence of varices			0.356	<0.001			0.024	0.818
Present	145 (75.9)	169 (59.5)			124.0 (63.9)	183.3 (65.1)		
Absent	46 (24.1)	115 (40.5)			70.0 (36.1)	98.4 (34.9)		
Extrahepatic metastasis			0.282	0.003			0.018	0.854
Present	106 (55.5)	196 (69.0)			126.4 (65.2)	181.1 (64.3)		
Absent	85 (44.5)	88 (31.0)			67.6 (34.8)	100.5 (35.7)		
Maximal tumor size			0.047	0.613			0.015	0.881
≤10 cm	93 (48.7)	145 (51.1)			99.6 (51.4)	142.5 (50.6)		
>10 cm	98 (51.3)	139 (48.9)			94.4 (48.6)	139.1 (49.4)		
Number of tumors			0.018	0.843			0.013	0.897
<4	118 (61.8)	178 (62.7)			117.6 (60.6)	172.6 (61.3)		
≥4	73 (38.2)	106 (37.3)			76.4 (39.4)	109.1 (38.7)		
AFP			0.014	0.877			0.017	0.867
< 400 ng/mL	76 (39.8)	111 (39.1)			72.8 (37.5)	107.9 (38.3)		
≥400 ng/mL	115 (60.2)	173 (60.9)			121.3 (62.5)	173.8 (61.7)		
Tumor type			0.143	0.126			0.014	0.889
Nodular	84 (44.0)	105 (37.0)			75.1 (38.7)	111.0 (39.4)		
Infiltrative	107 (56.0)	179 (63.0)			118.9 (61.3)	170.7 (60.6)		
Main PV or IVC invasion			0.129	0.17			<0.001	0.998
Present	107 (56.0)	177 (62.3)			115.9 (59.7)	168.2 (59.7)		
Absent	84 (44.0)	107 (37.7)			78.1 (40.3)	113.5 (40.3)		

* *p* values were calculated using IPTW-weighted Cox regression. ASD: Absolute standardized difference (<0.1 indicates adequate balance).

**Table 2:** Hazard ratios for 3-year clinical outcomes comparing Atezolizumab–Bevacizumab with locoregional therapy.

		Unadjusted Sample		IPTW-Weighted Sample	
	Treatment	HR (95% CI)	*p*	HR (95% CI) *	*p*
OS	Locoregional therapy	Ref		Ref	
	Atezolizumab–Bevacizumab	1.03 (0.84–1.26)	0.794	0.95 (0.76–1.19)	0.635
PFS	Locoregional therapy	Ref		Ref	
	Atezolizumab–Bevacizumab	0.70 (0.58–0.86)	<0.001	0.64 (0.52–0.79)	<0.001

* An inverse probability-weighted Cox proportional hazards regression model was used.

**Table 3:** Frequencies of secondary outcomes.

		Unadjusted Sample			IPTW-Weighted Sample		
		Atezolizumab–Bevacizumab (*n* = 191)	Locoregional Therapy (*n* = 284)	*p*	Atezolizumab–Bevacizumab (*n* = 194)	Locoregional Therapy (*n* = 282)	*p*
Objective response	Responder	86 (45.0)	137 (48.2)	0.491	92.5 (47.7)	135.1 (47.9)	0.957
	Non-responder	105 (55.0)	147 (51.8)		101.5 (52.3)	146.6 (52.1)	
Major adverse event	Present	170 (89.0)	252 (88.7)	0.926	172.0 (88.6)	249.6 (88.6)	0.993
	Absent	21 (11.0)	32 (11.3)		22.1 (11.4)	32.1 (11.4)	

## Data Availability

The data that support the findings of this study are available from the corresponding author upon reasonable request.

## Supplementary Materials

The following supporting information can be downloaded at https://www.mdpi.com/article/10.3390/cancers18010033/s1, Table S1: Event frequencies and Kaplan–Meier estimates of clinical outcomes before and after IPTW.

## Abbreviations

The following abbreviations are used in this manuscript:

HCC	Hepatocellular carcinoma
MVI	Macrovascular invasion
BCLC	Barcelona Clinic Liver Cancer
Atezo–Bev	Atezolizumab plus bevacizumab
OS	Overall survival
PFS	Progression-free survival
TACE	Transarterial chemoembolization
RT	External-beam radiotherapy/Radiotherapy
PVTT	Portal vein tumor thrombosis
IPTW	Inverse probability of treatment weighting
ECOG	Eastern Cooperative Oncology Group
CT	Computed tomography
MRI	Magnetic resonance imaging
mRECIST	Modified Response Evaluation Criteria in Solid Tumors
CR	Complete response
PR	Partial response
SD	Stable disease
PD	Progressive disease
HR	Hazard ratio
CI	Confidence interval
AFP	Alpha-fetoprotein
IQR	Interquartile range
GTV	Gross tumor volume
ITV	Internal target volume
PTV	Planning target volume
TARE	Transarterial radioembolization
TKI	Tyrosine kinase inhibitor
DNA-PK	DNA-dependent protein kinase

## References

[1] Sung H., Ferlay J., Siegel R.L., Laversanne M., Soerjomataram I., Jemal A., Bray F. (2021). Global Cancer Statistics 2020: GLOBOCAN Estimates of Incidence and Mortality Worldwide for 36 Cancers in 185 Countries. CA Cancer J. Clin..

[2] Villanueva A. (2019). Hepatocellular Carcinoma. N. Engl. J. Med..

[3] Vogel A., Rimassa L., Sun H.C., Abou-Alfa G.K., El-Khoueiry A., Pinato D.J., Sanchez Alvarez J., Daigl M., Orfanos P., Leibfried M. (2021). Comparative Efficacy of Atezolizumab plus Bevacizumab and Other Treatment Options for Patients with Unresectable Hepatocellular Carcinoma: A Network Meta-Analysis. Liver Cancer.

[4] Reig M., Forner A., Rimola J., Ferrer-Fàbrega J., Burrel M., Garcia-Criado Á., Kelley R.K., Galle P.R., Mazzaferro V., Salem R. (2022). BCLC strategy for prognosis prediction and treatment recommendation: The 2022 update. J. Hepatol..

[5] Heimbach J.K., Kulik L.M., Finn R.S., Sirlin C.B., Abecassis M.M., Roberts L.R., Zhu A.X., Murad M.H., Marrero J.A. (2018). AASLD guidelines for the treatment of hepatocellular carcinoma. Hepatology.

[6] Finn R.S., Qin S., Ikeda M., Galle P.R., Ducreux M., Kim T.Y., Kudo M., Breder V., Merle P., Kaseb A.O. (2020). Atezolizumab plus Bevacizumab in Unresectable Hepatocellular Carcinoma. N. Engl. J. Med..

[7] Kudo M., Finn R.S., Galle P.R., Zhu A.X., Ducreux M., Cheng A.L., Ikeda M., Tsuchiya K., Aoki K.I., Jia J. (2023). IMbrave150: Efficacy and Safety of Atezolizumab plus Bevacizumab versus Sorafenib in Patients with Barcelona Clinic Liver Cancer Stage B Unresectable Hepatocellular Carcinoma: An Exploratory Analysis of the Phase III Study. Liver Cancer.

[8] Cheng A.L., Qin S., Ikeda M., Galle P.R., Ducreux M., Kim T.Y., Lim H.Y., Kudo M., Breder V., Merle P. (2022). Updated efficacy and safety data from IMbrave150: Atezolizumab plus bevacizumab vs. sorafenib for unresectable hepatocellular carcinoma. J. Hepatol..

[9] Kulkarni A.V., Tevethia H., Kumar K., Premkumar M., Muttaiah M.D., Hiraoka A., Hatanaka T., Tada T., Kumada T., Kakizaki S. (2023). Effectiveness and safety of atezolizumab-bevacizumab in patients with unresectable hepatocellular carcinoma: A systematic review and meta-analysis. eClinicalMedicine.

[10] Song B.G., Goh M.J., Kang W., Sinn D.H., Gwak G.-Y., Choi M.S., Lee J.H., Paik Y.-H. (2024). Analysis of Factors Predicting the Real-World Efficacy of Atezolizumab and Bevacizumab in Patients with Advanced Hepatocellular Carcinoma. Gut Liver.

[11] Allaire M., Thiam E.M., Amaddeo G., Bouattour M., Edeline J., Brusset B., Ziol M., Merle P., Blanc J.F., Uguen T. (2025). Real-World Outcomes of Atezolizumab-Bevacizumab in Hepatocellular Carcinoma: The Prospective French CHIEF Cohort. Liver Int..

[12] Cheon J., Yoo C., Hong J.Y., Kim H.S., Lee D.W., Lee M.A., Kim J.W., Kim I., Oh S.B., Hwang J.E. (2022). Efficacy and safety of atezolizumab plus bevacizumab in Korean patients with advanced hepatocellular carcinoma. Liver Int..

[13] Manfredi G.F., Fulgenzi C.A.M., Celsa C., Stefanini B., D’Alessio A., Pinter M., Scheiner B., Awosika N., Brunetti L., Lombardi P. (2025). Efficacy of atezolizumab plus bevacizumab for unresectable HCC: Systematic review and meta-analysis of real-world evidence. JHEP Rep..

[14] Yoon S.M., Ryoo B.Y., Lee S.J., Kim J.H., Shin J.H., An J.H., Lee H.C., Lim Y.S. (2018). Efficacy and Safety of Transarterial Chemoembolization Plus External Beam Radiotherapy vs Sorafenib in Hepatocellular Carcinoma with Macroscopic Vascular Invasion: A Randomized Clinical Trial. JAMA Oncol..

[15] Goh M.J., Sinn D.H., Kim J.M., Lee M.W., Hyun D.H., Yu J.I., Hong J.Y., Choi M.S. (2023). Clinical practice guideline and real-life practice in hepatocellular carcinoma: A Korean perspective. Clin. Mol. Hepatol..

[16] Lee S.K., Kwon J.H., Lee S.W., Lee H.L., Kim H.Y., Kim C.W., Song D.S., Chang U.I., Yang J.M., Nam S.W. (2023). A Real-World Comparative Analysis of Atezolizumab Plus Bevacizumab and Transarterial Chemoembolization Plus Radiotherapy in Hepatocellular Carcinoma Patients with Portal Vein Tumor Thrombosis. Cancers.

[17] Marrero J.A., Kulik L.M., Sirlin C.B., Zhu A.X., Finn R.S., Abecassis M.M., Roberts L.R., Heimbach J.K. (2018). Diagnosis, Staging, and Management of Hepatocellular Carcinoma: 2018 Practice Guidance by the American Association for the Study of Liver Diseases. Hepatology.

[18] Sangro B., Argemi J., Ronot M., Paradis V., Meyer T., Mazzaferro V., Jepsen P., Golfieri R., Galle P., Dawson L. (2025). EASL Clinical Practice Guidelines on the management of hepatocellular carcinoma. J. Hepatol..

[19] Persano M., Rimini M., Tada T., Suda G., Shimose S., Kudo M., Rossari F., Yoo C., Cheon J., Finkelmeier F. (2024). Adverse Events as Potential Predictive Factors of Activity in Patients with Advanced HCC Treated with Atezolizumab Plus Bevacizumab. Target. Oncol..

[20] Baerlocher M.O., Nikolic B., Sze D.Y. (2023). Adverse Event Classification: Clarification and Validation of the Society of Interventional Radiology Specialty-Specific System. J. Vasc. Interv. Radiol..

[21] Kim J.H., Shim J.H., Yoon H.K., Ko H.K., Kim J.W., Gwon D.I. (2018). Chemoembolization related to good survival for selected patients with hepatocellular carcinoma invading segmental portal vein. Liver Int..

[22] Jung J., Joo J.H., Kim S.Y., Kim J.H., Choi J., Lee D., Shim J.H., Kim K.M., Lim Y.S., Lee H.C. (2022). Radiologic Response as a Prognostic Factor in Advanced Hepatocellular Carcinoma with Macroscopic Vascular Invasion after Transarterial Chemoembolization and Radiotherapy. Liver Cancer.

[23] Kim Y.J., Jung J., Joo J.H., Kim S.Y., Kim J.H., Lim Y.S., Lee H.C., Kim J.H., Yoon S.M. (2019). Combined transarterial chemoembolization and radiotherapy as a first-line treatment for hepatocellular carcinoma with macroscopic vascular invasion: Necessity to subclassify Barcelona Clinic Liver Cancer stage C. Radiother. Oncol..

[24] Lencioni R., Llovet J.M. (2010). Modified RECIST (mRECIST) assessment for hepatocellular carcinoma. Semin. Liver Dis..

[25] Lencioni R., Montal R., Torres F., Park J.W., Decaens T., Raoul J.L., Kudo M., Chang C., Ríos J., Boige V. (2017). Objective response by mRECIST as a predictor and potential surrogate end-point of overall survival in advanced HCC. J. Hepatol..

[26] Park C., Chu H.H., Kim J.H., Kim S.Y., Alrashidi I., Gwon D.I., Yoon H.K., Kim N. (2020). Clinical Significance of the Initial and Best Responses after Chemoembolization in the Treatment of Intermediate-Stage Hepatocellular Carcinoma with Preserved Liver Function. J. Vasc. Interv. Radiol..

[27] Lyu N., Wang X., Li J.B., Lai J.F., Chen Q.F., Li S.L., Deng H.J., He M., Mu L.W., Zhao M. (2022). Arterial Chemotherapy of Oxaliplatin Plus Fluorouracil Versus Sorafenib in Advanced Hepatocellular Carcinoma: A Biomolecular Exploratory, Randomized, Phase III Trial (FOHAIC-1). J. Clin. Oncol..

[28] Park Y., Cho Y., Kim S.U., Kim A., Shin H., Kim H.C., Lee I.J., Kim G.M., Hyun D., Ko Y. (2025). Transarterial radioembolization versus atezolizumab-bevacizumab for the treatment of hepatocellular carcinoma with portal vein tumor thrombosis. Diagn. Interv. Imaging.

[29] Kim G.H., Kim J.H., Kim P.H., Chu H.H., Gwon D.I., Ko H.-K. (2021). Emerging Trends in the Treatment of Advanced Hepatocellular Carcinoma: A Radiological Perspective. Korean J. Radiol..

[30] Amir E., Seruga B., Kwong R., Tannock I.F., Ocaña A. (2012). Poor correlation between progression-free and overall survival in modern clinical trials: Are composite endpoints the answer?. Eur. J. Cancer.

[31] Hwang H., Shim J.H., Kim J.H. (2024). Hepatocellular carcinoma with macrovascular invasion: Need a personalized medicine for this complicated event. Hepatobiliary Surg. Nutr..

[32] Chen Z.H., Wang K., Zhang X.P., Feng J.K., Chai Z.T., Guo W.X., Shi J., Wu M.C., Lau W.Y., Cheng S.Q. (2020). A new classification for hepatocellular carcinoma with hepatic vein tumor thrombus. Hepatobiliary Surg. Nutr..

[33] Boeckman H.J., Trego K.S., Turchi J.J. (2005). Cisplatin sensitizes cancer cells to ionizing radiation via inhibition of nonhomologous end joining. Mol. Cancer Res..

[34] Lu J., Zhao M., Arai Y., Zhong B.Y., Zhu H.D., Qi X.L., de Baere T., Pua U., Yoon H.K., Madoff D.C. (2021). Clinical practice of transarterial chemoembolization for hepatocellular carcinoma: Consensus statement from an international expert panel of International Society of Multidisciplinary Interventional Oncology (ISMIO). Hepatobiliary Surg. Nutr..

[35] Ha Y., Kim J.H., Cheon J., Jeon G.S., Kim C., Chon H.J. (2023). Risk of Variceal Bleeding in Patients with Advanced Hepatocellular Carcinoma Receiving Atezolizumab/Bevacizumab. Clin. Gastroenterol. Hepatol..

[36] Thornton L.M., Abi-Jaoudeh N., Lim H.J., Malagari K., Spieler B.O., Kudo M., Finn R.S., Lencioni R., White S.B., Kokabi N. (2024). Combination and Optimal Sequencing of Systemic and Locoregional Therapies in Hepatocellular Carcinoma: Proceedings from the Society of Interventional Radiology Foundation Research Consensus Panel. J. Vasc. Interv. Radiol..

[37] Agirrezabal I., Bouattour M., Pinato D.J., D’Alessio A., Brennan V.K., Carion P.L., Shergill S., Amoury N., Vilgrain V. (2024). Efficacy of transarterial radioembolization using Y-90 resin microspheres versus atezolizumab-bevacizumab in unresectable hepatocellular carcinoma: A matching-adjusted indirect comparison. Eur. J. Cancer.

[38] Kim J.H., Kim G.H., Gwon D.I. (2024). Reappraisal of transarterial radioembolization for liver-confined hepatocellular carcinoma with portal vein tumor thrombosis: Editorial on “Transarterial radioembolization versus tyrosine kinase inhibitor in hepatocellular carcinoma with portal vein thrombosis”. Clin. Mol. Hepatol..

[39] Vilgrain V., Pereira H., Assenat E., Guiu B., Ilonca A.D., Pageaux G.P., Sibert A., Bouattour M., Lebtahi R., Allaham W. (2017). Efficacy and safety of selective internal radiotherapy with yttrium-90 resin microspheres compared with sorafenib in locally advanced and inoperable hepatocellular carcinoma (SARAH): An open-label randomised controlled phase 3 trial. Lancet Oncol..

[40] Chow P.K.H., Gandhi M., Tan S.B., Khin M.W., Khasbazar A., Ong J., Choo S.P., Cheow P.C., Chotipanich C., Lim K. (2018). SIRveNIB: Selective Internal Radiation Therapy Versus Sorafenib in Asia-Pacific Patients with Hepatocellular Carcinoma. J. Clin. Oncol..

[41] Garin E., Tselikas L., Guiu B., Chalaye J., Edeline J., de Baere T., Assenat E., Tacher V., Robert C., Terroir-Cassou-Mounat M. (2021). Personalised versus standard dosimetry approach of selective internal radiation therapy in patients with locally advanced hepatocellular carcinoma (DOSISPHERE-01): A randomised, multicentre, open-label phase 2 trial. Lancet Gastroenterol. Hepatol..

[42] Casak S.J., Donoghue M., Fashoyin-Aje L., Jiang X., Rodriguez L., Shen Y.L., Xu Y., Jiang X., Liu J., Zhao H. (2021). FDA Approval Summary: Atezolizumab Plus Bevacizumab for the Treatment of Patients with Advanced Unresectable or Metastatic Hepatocellular Carcinoma. Clin. Cancer Res..

