# Supplementary material for: Atezolizumab Plus Bevacizumab for Advanced Hepatocellular Carcinoma with Macroscopic Vascular Invasion: An Inverse Probability of Treatment Weighted Analysis
Source: Cancers (Basel). 2025 Dec 22;18(1):33. doi: 10.3390/cancers18010033 (PMC12784712; doi:10.3390/cancers18010033)
Supplement: Supplementary file 1 [file cancers-18-00033-s001.zip › cancers-4027868-supplementary.pdf]

Table S1. Event frequencies and Kaplan-Meier estimates of clinical outcomes before and after IPTW.

		**Unadjusted sample**				**IPTW-weighted sample**			
	Follow-up time*	Overall (n=475)	Atezolizumab-Bevacizumab (n=191)	Locoregional Therapy (n=284)	p*	Overall (n=476)	Atezolizumab-Bevacizumab (n=194)	Locoregional Therapy (n=282)	p**
OS	12	259 (55.2%)	106 (55.6%)	153 (55.0%)	0.638	252 (53.6%)	100 (51.4%)	153 (55.2%)	0.6
	24	362 (80.1%)	141 (78.8%)	221 (80.5%)	0.791	361 (79.9%)	140 (77.7%)	221 (81.0%)	0.662
	36	386 (87.4%)	146 (84.7%)	240 (87.7%)	0.794	385 (87.2%)	145 (83.2%)	240 (88.1%)	0.635
	48	397 (91.5%)	146 (84.7%)	251 (91.9%)	0.851	395 (91.6%)	145 (83.2%)	251 (92.4%)	0.606
	60	405 (95.0%)		259 (95.3%)	NA	403 (95.3%)		258 (95.7%)	NA
	72	405 (95.0%)		259 (95.3%)	NA	403 (95.3%)		258 (95.7%)	NA
	84	407 (97.5%)		261 (97.7%)	NA	405 (97.4%)		260 (97.6%)	NA
PFS	12	368 (78.9%)	135 (70.9%)	233 (84.5%)	.<001	366 (78.3%)	133 (69.0%)	233 (84.9%)	<.001
	24	416 (91.4%)	159 (87.2%)	257 (94.4%)	.<001	415 (91.2%)	159 (86.1%)	257 (94.9%)	<.001
	36	419 (92.9%)	160 (89.8%)	259 (95.4%)	.<001	419 (93.3%)	160 (91.1%)	259 (95.8%)	<.001
	48	422 (94.9%)	160 (89.8%)	262 (96.8%)	.<001	422 (95.4%)	160 (91.1%)	262 (97.3%)	<.001
	60	424 (96.5%)		264 (97.9%)	NA	424 (96.9%)		263 (98.2%)	NA
	72	424 (96.5%)		264 (97.9%)	NA	424 (96.9%)		263 (98.2%)	NA
	84	425 (100.0%)		265 (100.0%)	NA	424 (100.0%)		264 (100.0%)	NA

*Log-rank test was used.

**Wald-test with robust variance estimation was used.
